# The impact of injury on apparent survival of whale sharks (*Rhincodon typus*) in South Ari Atoll Marine Protected Area, Maldives

**DOI:** 10.1038/s41598-020-79101-8

**Published:** 2021-01-13

**Authors:** Jessica Harvey-Carroll, Joshua D. Stewart, Daire Carroll, Basith Mohamed, Ibrahim Shameel, Irthisham H. Zareer, Gonzalo Araujo, Richard Rees

**Affiliations:** 1 Maldives Whale Shark Research Programme (MWSRP), South Ari Atoll, Maldives; 2 School of Psychology and Neuroscience, University of St Andrews, St Andrews, UK; 3 The Manta Trust, Dorchester, UK; 4 The James Hutton Institute, Dundee, UK; 5 The University of Warwick, School of Life Science, Coventry, UK; 6 Large Marine Vertebrates Research Institute Philippines, Cagulada Compound, 6308 Jagna, Bohol Philippines

**Keywords:** Ecology, Ecological modelling, Population dynamics

## Abstract

The whale shark (*Rhincodon typus*) is an endangered species with a declining global population. The South Ari Atoll Marine Protected Area (SAMPA), Maldives, is one of few locations globally where year-long residency of individuals occurs. This SAMPA aggregation appears to consist almost exclusively of immature males. Due to its year-round residency, this local aggregation is subjected to a high degree of tourism pressure. This ecotourism contributes to the high level of interest and protection offered to whale sharks by the local community. Unfortunately, if regulations are not followed or enforced, tourism can bring with it major stressors, such as accidental injuries. We used POPAN capture-mark-recapture models and lagged identification rate analysis to assess the effect of major injuries on whale shark residency within SAMPA. Injuries may be obtained outside SAMPA. We found individuals with major injuries had a higher apparent survival in the area than those without. Lagged identification rates also demonstrated that sharks with major injuries are more likely to return to the area. We suggest that major injuries result in sharks prolonging their time in the developmental habitat. These findings have implications for individual fitness and the population viability of this endangered species. We propose targeted conservation strategies be considered to protect sharks from further injury. Based on the presented spatio-temporal distributions of sharks, and current local knowledge of sighting patterns, speed limit zones and propeller-exclusion zones should be implemented and enforced. If carried out alongside tourist education, these measures will contribute to the protection of whale sharks within SAMPA and beyond. Furthermore, our results can aid research direction, alongside regulation and enforcement development, at similar sites worldwide.

## Introduction

Pressure on marine life is increasing, with marine megafauna being heavily impacted by human activities^1^. Overfishing, habitat destruction, and climate change are among the anthropogenic threats directly responsible for marine biodiversity loss and species declines^2^. Sharks and rays often exhibit long lifespans, late maturity, and slow growth^3,4^. These life history traits make them especially susceptible to overexploitation^5,6^ and subsequent population decline^2^. Sharks frequently use areas with abundant food and protection for juveniles as nurseries, or developmental habitats^7^. This life trait is similar to that observed in the early development of other marine megafauna (e.g. marine turtles^8^). Such nurseries often overlap with areas of frequent human activity, such as valuable coastal fishing grounds. This overlap puts nurseries at particular risk of anthropogenic pressures and ecosystem disruption, which can affect shark populations^9,10^. Human activity, such as improperly managed tourism, can negatively alter shark behaviour, metabolism and species distribution^11^. Despite local and national implementation of protected marine regions, and a multitude of other regulations (such as finning bans, catch limits, and traditional fisheries management approaches) global shark populations continue to decline^12^. Marine Protected Areas (MPAs) have been highlighted as a possible effective management tools for sharks^12^, yet poor enforcement reduces or negates the effectiveness of MPAs in many regions^13^.

The whale shark (*Rhincodon typus*) is the world’s largest shark species. Their conservation status has been uplisted to Endangered in the IUCN Red List of Threatened Species^14^. Despite the public attention received by this charismatic species, knowledge gaps remain about anthropogenic impacts on populations. Conservation efforts over the last three decades have led to legal protection for the species on an international scale. Whale sharks are now listed in Appendix II of CITES^15^ and Appendices I and II of CMS^16^, which are major stepping stones in protecting shark populations^17^. The benefits of protection are likely to take several years to occur and currently populations continue to decline, for example in Mozambique^14,18^. Given documented exploitation of whale sharks in the indo-pacific^19,20^, understanding the impacts of anthropogenic activities on the species is important for directing future conservation efforts.

Whale sharks are highly mobile, with some individuals travelling thousands of kilometres in a few months^21^. These large-scale movements can often involve multiple political jurisdictions^22–25^. As a result, aggregations of whale sharks within MPA’s are still influenced by human activities and fishing elsewhere^26^. The highly mobile nature of whale sharks highlights the need to implement a cohesive global plan for protection^27,28^. Whale sharks are thought to display seasonal philopatry^29,30^, with large annual aggregations occurring globally^31,32^. The biological functions of these aggregations still warrant further investigation.

The South Ari Atoll Marine Protected Area (SAMPA), in the Maldives, features a year-round local aggregation of whale sharks^33,34^. Continuous presence of the same individuals in one area is only seen in a few sites globally^35–37^. SAMPA is thought to act as a developmental habitat for whale sharks—an area characterized by immature individuals displaying strong site fidelity^8^. The SAMPA aggregation consists of juvenile individuals (averaging 5 m in length)^38^, and is thought to provide food and protection from predators. The survival of older juveniles is thought to have more influence on shark and ray population stability, and recovery, than neonates^10^. Whale sharks gained protection from the Maldivian government in 1995^39^. The Maldivian shark fishing industry has since been replaced with ecotourism and provides an important source of income in the Maldives^40^. This, in combination with the juvenile composition of SAMPA, highlights the importance of assessing the status of this aggregation. SAMPA whale shark population dynamics and the effects of injuries from intensive ecotourism and vessel traffic has not yet been assessed. At sites with year-round residency and high philopatry, such as SAMPA, it is currently unclear how injury might affect residency and visitation patterns.

Capture mark recapture (CMR) studies provide an ideal methodology for studying whale shark populations. Whale sharks have unique markings on their bodies which can be used for individual identification and validated through pattern-recognition software^41^. Photo-ID data can be used for CMR modelling, as has been done at Ningaloo Marine Park in Western Australia, The Galapagos Marine Reserve, and the Seychelles^42–45^. In this study, we use CMR analysis to investigate whale shark population dynamics within SAMPA. Photo identification data was generated by Maldives Whale Shark Research Programme (MWSRP) and citizen scientist contributors between 2006 and 2019. We investigate the effects of major injuries on the residency and apparent survival (which does not distinguish between death and permanent emigration) of whale sharks in SAMPA using two complementary methods: a POPAN Jolly-Seber model^46,47^ and lagged identification rate analysis (LIR)^48^.

## Materials and methods

### Study area and surveys

**Figure 1:**
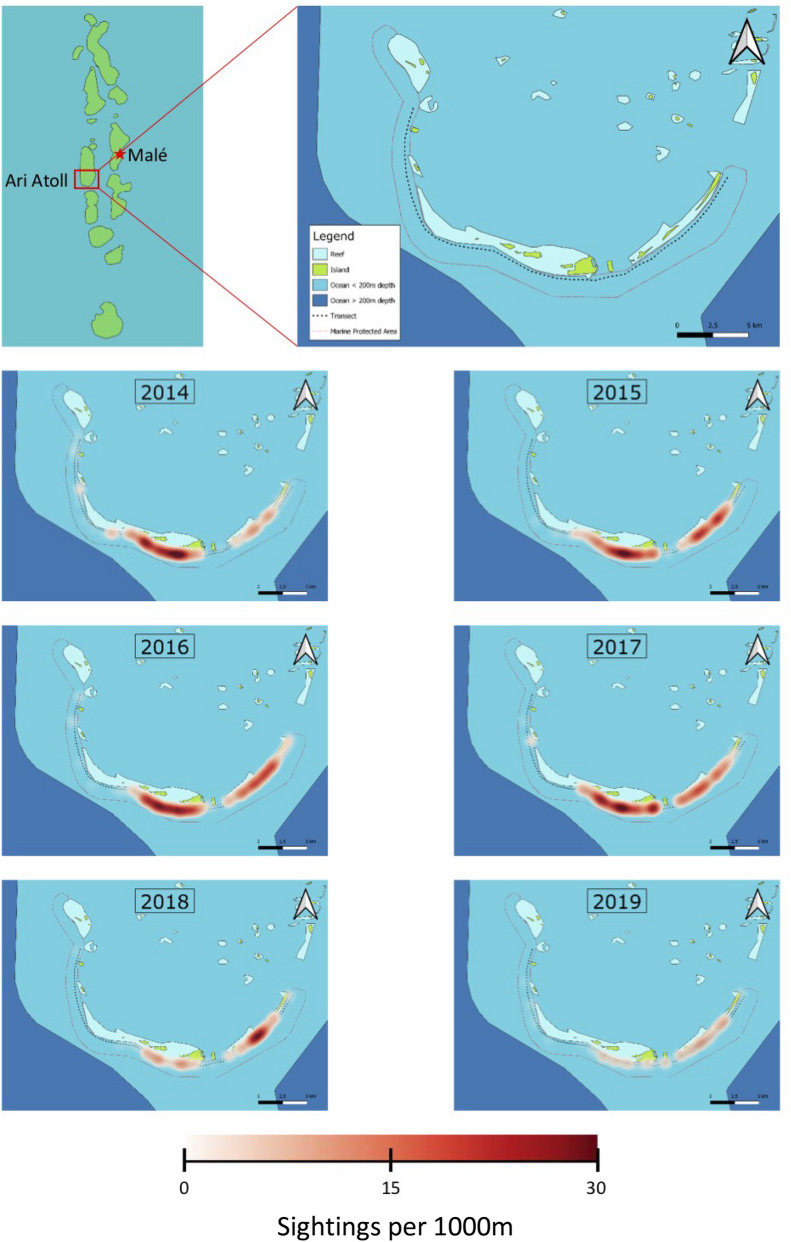
The South Ari Atoll MPA showing Whale shark sightings recorded by MWSRP during routine survey work in SAMPA between the years 2014 and 2019 as heatmaps. Sighting numbers saw a steady decline with 245 sightings in 2014, 268 in 2015, 300 in 2016, 295 in 2017, 149 in 2018 and 103 in 2019. Maps were created using QGIS^49^.

MWSRP carried out surveys as per Riley et al.^34^, along the Maamigili-Dhigurah Reef (03° 28′ N, 72° 51′ E) (Fig. 1). Upon sighting of a shark, trained MWSRP staff entered the water and collected photo-identification and sex data. Sex was determined by the presence (male) or absence (female) of claspers between the pelvic fins^34^. MWSRP staff parallel to the shark recorded length using a tape measure. They also recorded distinguishing features, such as injuries (Fig. 1). Surveys were carried out 5 days a week (Sunday–Thursday), weather depending. Surveys were conducted using consistent methodology, the same survey platform (boat) across years, and at least two trained researchers. Surveys prior to 2015 departed from Conrad, went to Dhigurah and back again. From 2015 onwards, surveys departed from Dhigurah, heading to Conrad and back again. MWSRP were unable to survey the full extent of the MPA every day due to external circumstances such as time, weather, or logistical constraints.

The Big Fish Network is an online portal for citizen scientists to submit whale shark encounters in the Maldives. Contributors to the Big Fish Network are trained by MWSRP to record information on whale shark encounters and photo-identify them. Upon submission of an encounter, MWSRP verifies the submission and approves the data collected. This information is then recorded in the MWSRP dataset.

We generated heatmaps to visualize yearly sightings by MWSRP surveys and represent the density of sightings along the transect during each survey season (2014–2019). We created maps using QGIS^49^. We calculated the density of sightings along the transect during each survey season (2014–2019) using quadratic kernel density estimation with a radius of 1000 m.

### Individual identification

We used I3S classic version 4.02 to identify sharks based on photographs of the left flank of the shark. If an imagine from the left was not obtained, a right flank image was used^50^. This software displays the 10 closest matches for each image. MWSRP staff then made the final identification manually from the 10 returned sharks. We took unmatched sharks to be potential new individuals. We subjected these to checks by at least two other MWSRP staff members. MWSRP staff also carried a manual search of the entire register before confirming each ‘new’ individual.

### Injury categories

**Figure 2:**
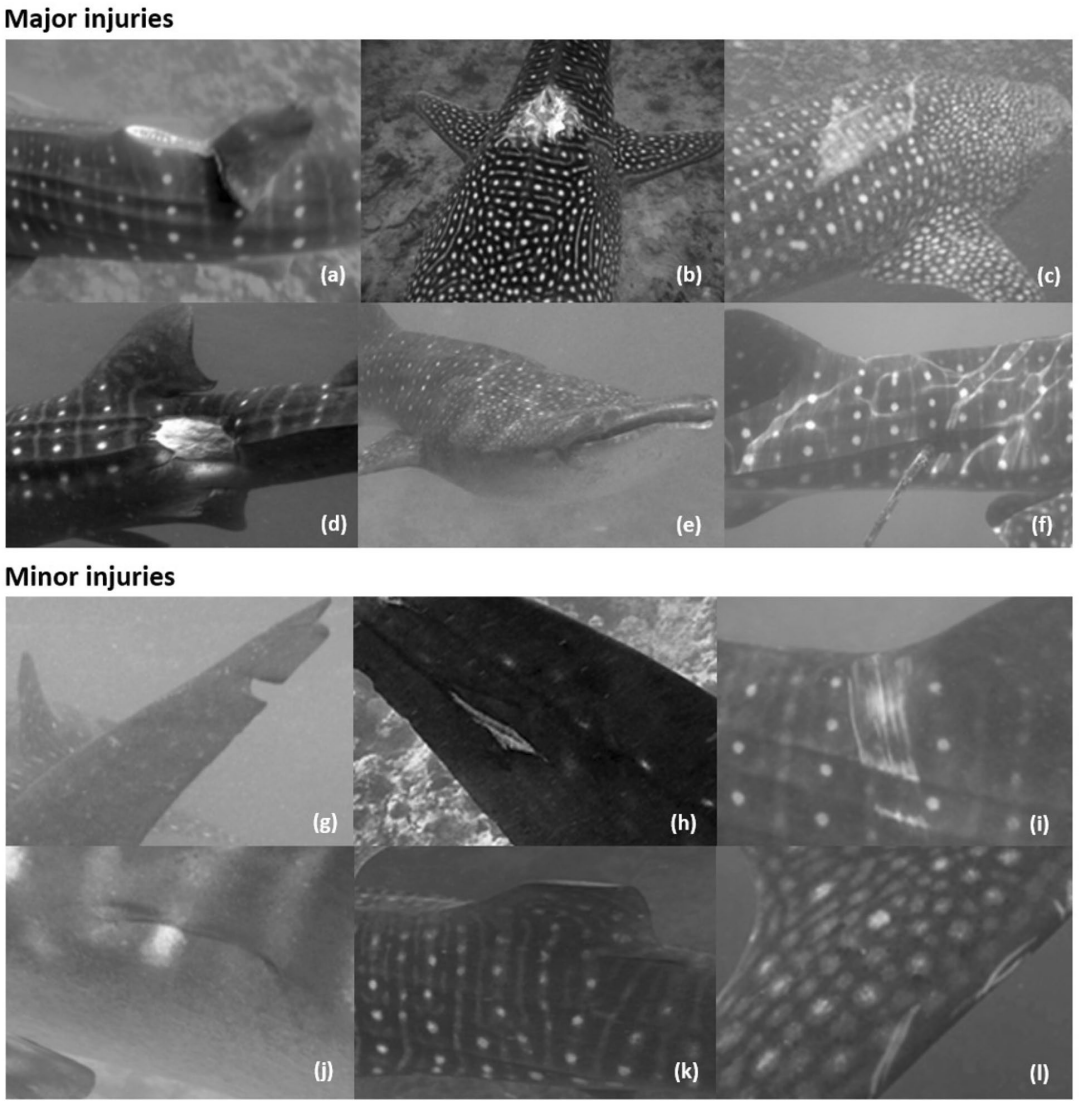
Injury classification examples based on Speed et al.^27^: Major injuries (**a**) amputation, (**b**) laceration, (**c**) abrasion, (**d**) bites, (**e**) blunt trauma, (**f**) impalement. Entanglement not shown. Minor injuries—(**g**) amputation, (**h**) laceration, (**i**) abrasion (penetrating outer dermal layer), (**j**) bites, (**k**) deformity and (**l**) scratch. Images taken by MWSRP.

We categorised injuries as either major or minor, and new or old. New injuries indicate that the injury had not previously been seen on an individual. New injuries also typically have exposed subdermal tissue, however, there is currently no definitive way to measure age of the injury. Injury classification was based on Speed et al.^27^ (Fig. 2). Minor injuries are generally thought of as superficial, causing minimal damage^27^. We did not include these in further analyses. Major injuries are thought to be potentially life-threatening—typically including dorsal, caudal or pectoral fin amputations and damage penetrating to at least the sub-dermal layer^27,28^. For all analysis sharks were grouped as injured if they ever had a major injury, as per Lester et al.^28^. Within the SAMPA aggregation, propeller collisions are believed to be the main causes of major injuries (lacerations and amputations).

### Data processing for capture mark recapture modelling

Data processing, analysis, and modelling was carried out using R^51^.

We assessed the homogeneity of survey dates each year by comparison of survey frequencies per month. Sampling data prior to 2014 was highly variable in search effort and consistency, violating Jolly-Seber assumptions. We therefore selected the years 2014–2019 for use within the CMR model.

Depending on weather conditions, survey months varied between years. All years had a total of 7 months surveying data with equal search effort. To maintain consistency, following POPAN assumptions of equal search effort, we excluded months during which weather conditions prevented routine surveying from POPAN analyses. Sampling consistently started in either January, February or March. Data was pooled by year, and aggregated yearly data was then analysed in the POPAN model. Transects were conducted in Jan–Mar, Aug–Nov in 2014; Feb–April, Aug–Nov in 2015, 2016, 2017 and 2019; and March, April, July–Nov in 2018.

### Modelling and data analysis

#### Capture mark recapture modelling

We fit a POPAN Jolly-Seber CMR model to the data using the R package *RMark*^52^. Robust design models were not used as the continuous survey design violated model assumptions of fixed closed and open periods of shark movement^53^.

We used POPAN models to estimate key population parameters. Apparent survival (ɸ) represented the probability of an animal surviving between times i and i + 1. Importantly, Jolly-Seber models cannot distinguish between death and permanent emigration in calculation of ɸ. Sighting probability (p) is the probability of an individual’s “capture” at any given time (t). Probability of entry (PENT) is the probability that a new animal will enter the population. The superpopulation size (N) represents all animals—both observed and unobserved—that are part of the population during the full study period.

We fit models which allowed ɸ, p, and PENT to remain constant or vary across time (t) and by sex. We added sex (male, female, or unknown) as a group covariate. We considered injuries as a group covariate for ɸ and p. The POPAN model does not allow for group covariates to vary across time^53^. We allowed N to vary by group or remain constant. We ran all biologically plausible combinations of parameter settings, producing 96 candidate models (Supplementary Table 1. Shows the top 20 models). We selected the most parsimonious candidate model based on lowest adjusted Akaike’s information criterion for small sample sizes (AICc’s) value, and in turn the ∆AICc^54^. There was a 1.4 AICc margin between the top and second model.

We derived abundance and gross population size from calculated POPAN parameter values. Gross population size (N*) is defined as the total number of all whale sharks available to be detected during the survey period, and sharks that enter and leave undetected between surveys.

#### CJS goodness of fit testing

In order to test that our data met the assumptions of a Jolly-Seber model we used *Test2* and *Test3* from the program RELEASE in the *RMark* package.

### Lagged identification rate

We used modified maximum likelihood methods, devised by Whitehead^48^, to estimate the lagged identification rate (LIR). LIR is defined as the probability that an individual will be resighted at the study site after a certain time lag. We used the program SOCPROG 2.9^55^ to estimate the LIR for whale sharks in SAMPA. We also produced estimates of residency, mortality or permanent emigration, and population size, for sharks with and without major injuries. The LIR uses the identification data itself to account for effort^48^ unlike CMR, and thus we were able to use data from 2006 to 2019 from MWSRP surveys and citizen science data from the Big Fish Network. We tested eight models (Supplementary Table 2) for goodness of fit. We used a combination of closed, open, emigration, mortality and reimmigration parameters as pre-set in the ‘Movement’ module of the program.

We separated the data to run the models for sharks with and without major injuries. We selected models based on the Quasi-Akaike information criterion (QAIC) to account for data overdispersion, with the best-fit models having a ΔQAIC of < 2^56^. The best fit-models were then bootstrapped for 100 repetitions to obtain confidence intervals (95%) and standard errors^57^.

### Testing for association between major injury on entry and residency

We carried out further analysis to determine the impact of major injuries upon entry to the SAMPA aggregation on returning status. We grouped individuals based on their injury status during their first MWSRP sighting between 2006 and 2019. We classified individuals with one or more major injuries during their first sighting as injured on entry. We classified those with no major injuries as non-injured on entry. We further categorized sharks into returning and non-returning groups. We defined returning sharks as those sighted on more than 1 year during MWSRP surveys between 2006 and 2019. Non-returning sharks were only sighted on a single year. New individuals sighted in 2019 were not included as returning status was unknown. We performed a chi-squared test for independence to test for a significant association between returning and major injury on entry status.

We also investigated the relationship between shark size (Total length (m)) on entry, major injury on entry, and residency status. We used the first recorded size measurement of each shark during an MSWRP encounter between 2006 and 2019 as its ‘size on entry’ into the aggregation. We carried out a two-way ANOVA to test for a significant difference in length between sharks grouped according to residency and major injury status. Following a significant result for the two-way ANOVA, we carried out a Tukey HSD post-hoc test.

### Ethics statement

All methods, including data collection surveys. were approved and carried out in accordance with the MWSRP Protected species research permit (A149508) from the Environmental protection agency and Ministry of Fisheries and Research permit (OTHR)30-D/PRIV/2019/1172 awarded by Ministry of Fisheries, Marine Resources and Agriculture, Male’, Maldives. No experimental protocols were used, as all work is based on survey data.

## Results

### Aggregation structure

**Figure 3:**
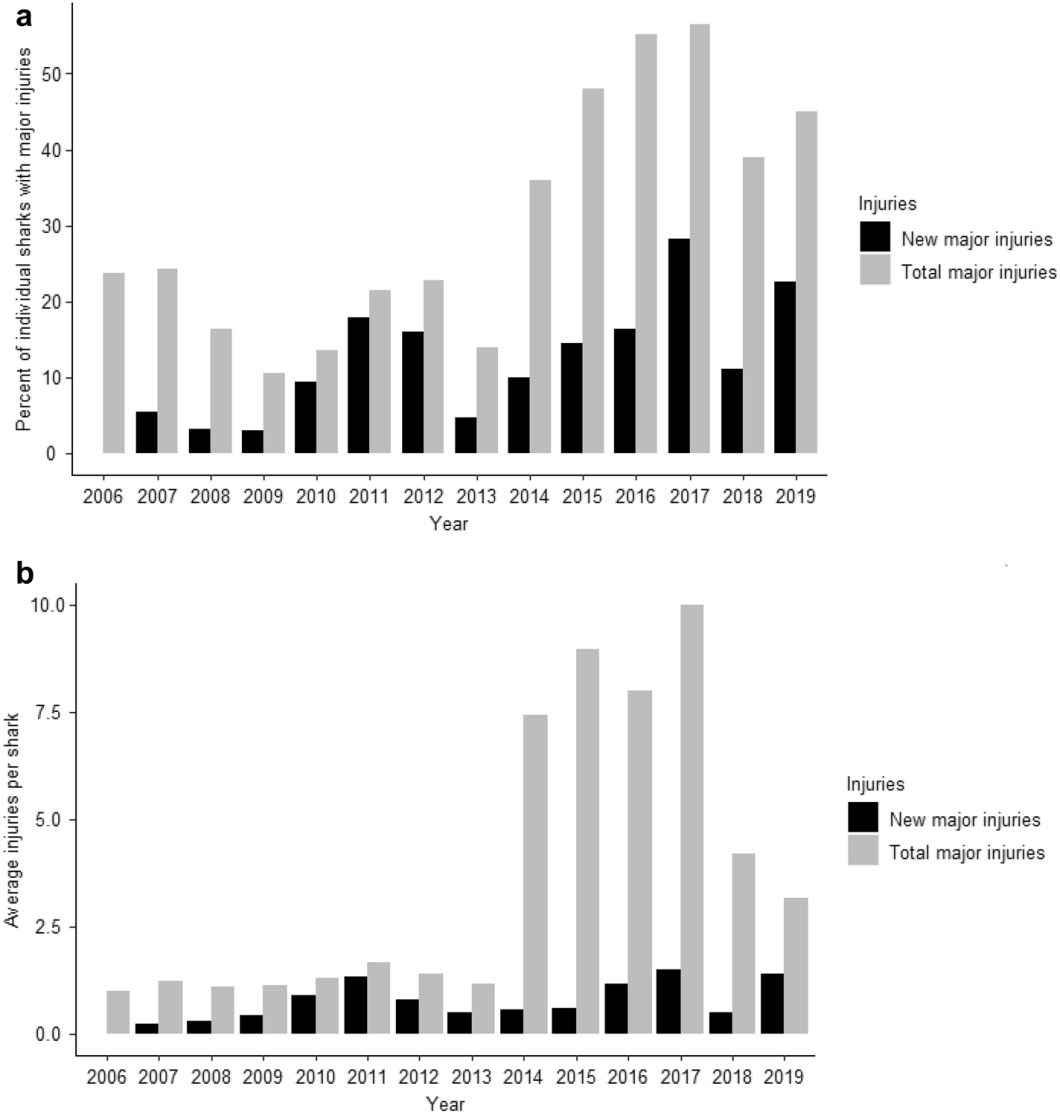
(**a**) Percentage of individual sharks from MWSRP data only sighted in a year with a new major injury (black) or any major injury (grey). (**b**) Average major injuries per injured shark. The maximum injuries observed on one individual was 49 during 2015.

For the entire dataset, including citizen science contributions, between the years 2006–2019, 265 unique individuals were recorded across 3445 sightings. A general increase of individual sharks with major injuries was present (Fig. 3a), alongside average injuries per shark (Fig. 3b). For the dataset used within the POPAN model, 100 unique individuals were recorded from 1479 sightings, between the years 2014–2019. Of unique individuals, 61 (61%) sharks were observed to have at least one major injury. Sex was determined for 93 individuals, of which 89 (95.69%) were male, and 4 (4.3%) were female. The highest number of sightings was recorded in 2016 (285) with the lowest number in 2019 (89) (Fig. 1).

**Figure 4:**
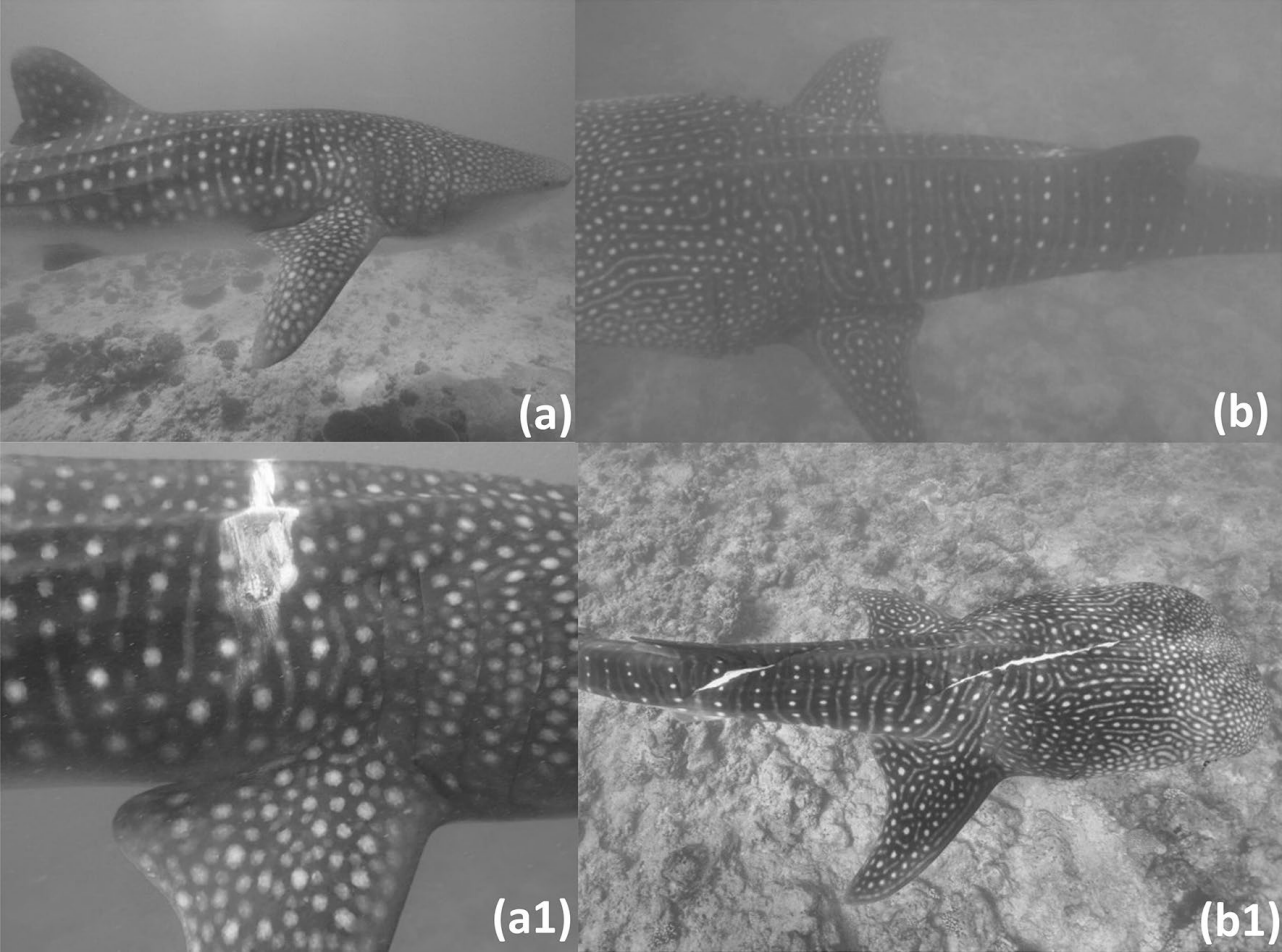
Example images of new major injuries obtained within estimated residency periods (**a**,**a1**) and outside of estimated residency periods (**b**,**b1**). This shark (WS180) was documented on 26.03.14 with no major injuries (**a**). A major impalement injury was present when the shark was next observed 6 days later on the 01.04.14 (**a1**). (**b**) This shark (WS077) was documented on 14.01.12 with no major injuries. (**b1**) The next sighting of WS077 (Photo credit: Big Fish Network) occurred 122 days later on the 15.05.12, and major lacerations were present. Images taken by MWSRP.

Using MWSRP survey data only, the average time between last sighting and first new major injury was calculated to be 128.65 days (range = 947) (Fig. 4).

### Mark recapture modelling

Goodness of fit testing indicating equal catchability (no trap dependence) was supported in TEST2 (P = 0.58, DF = 3, χ^2^ = 1.98). Test 3 indicated support of the Jolly-Seber assumption of equal survival probabilities to the following occasion (P = 0.72, DF = 10, χ^2^ =  7.09). The overall goodness of fit total was not significant (P = 0.77, DF = 13, χ^2^ =  9.06), indicating Jolly-Seber assumptions, including residency, were met by the data.

The most parsimonious model from the suite of candidate models demonstrated apparent survival (permanent emigration or death, ɸ) to be influenced by major injury and time (Supplementary Table 1). Sighting probability varied by presence of major injury and sex, whilst PENT varied by sex and the superpopulation size (N) remained constant. The presence of major injury grouping as a predictor of apparent survival (ɸ) was consistent amongst the top 54 models (Supplementary Table 1).

**Figure 5:**
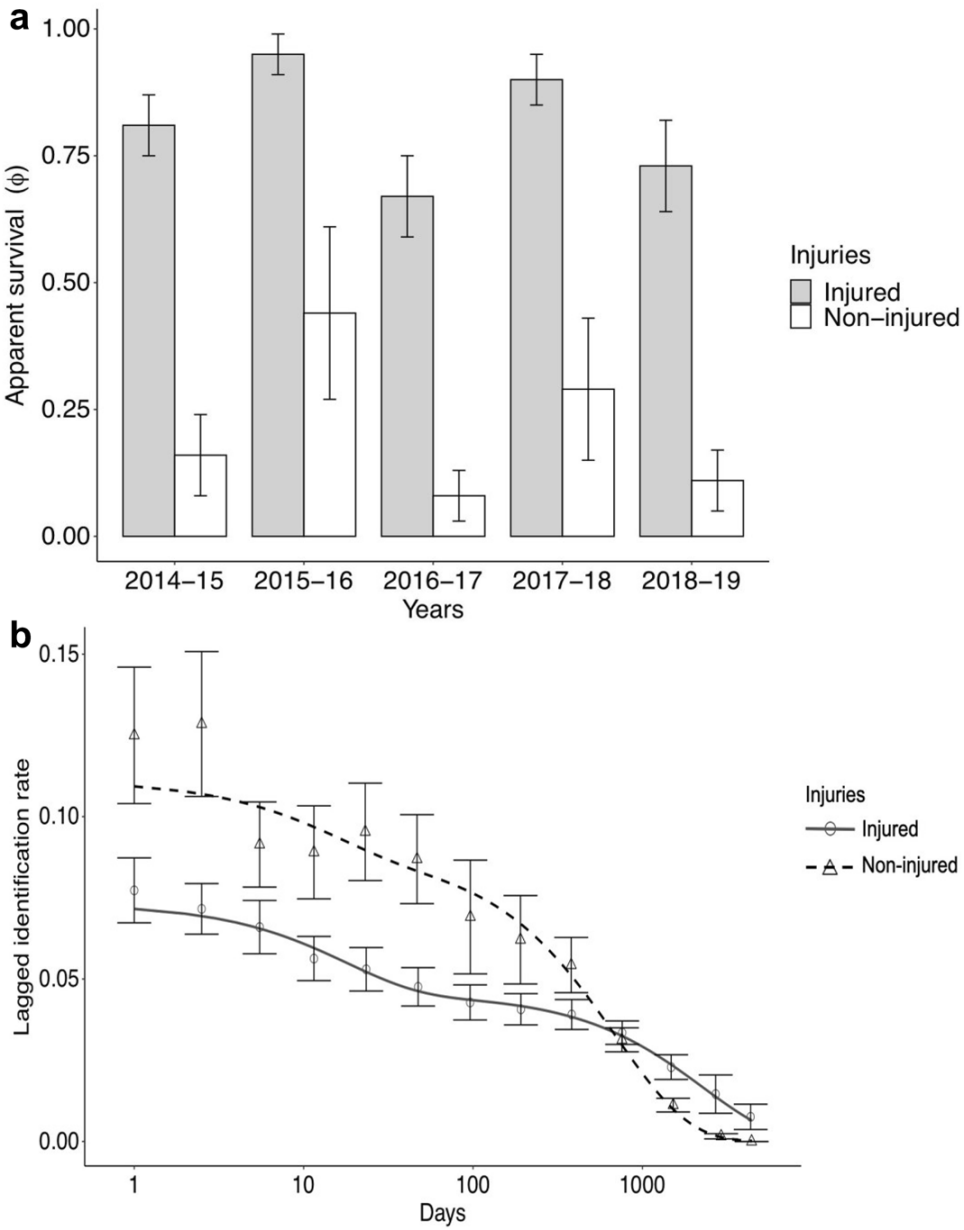
(**a**) Apparent survival (permanent emigration/death, with SE) between years i and i + 1 for whale sharks with major injuries (grey) and individuals with non-major injuries (white). (**b**) Lagged Identification Rate (with SE) for whale sharks in SAMPA with major injuries (grey, circles) and without (black, triangles).

The best-fit model demonstrated effects of major injury on ɸ across time. Sharks with major injuries were found to have a consistently higher ɸ than non-injured animals (higher ɸ likely representing lower emigration). No overlaps in the 95% confidence intervals were present between injured and non-injured sharks (Fig. 5a). It is likely that POPAN estimates for females could not be modelled accurately due to the small number in the dataset.

Sighting probability (p) for sharks with major injuries was estimated at 0.91 (SE = 0.03) for males and 0.55 (SE = 0.18) for females. We were unable to reliably model the sighting probability (p) of sharks with no major injuries (both males and females had a calculated value of 1.0, SE = 0). This is likely due to calculated sighting probability (p) being close to 1, causing them to become bounded. This is supported by the third most parsimonious model (Supplementary Table 1), in which sighting probability (p) is calculated based on injury alone. Sighting probability (p) for sharks with no major injuries was calculated to be 1.00 (SE = 7.89 × 10^–6^) and 0.88 for sharks with major injuries (SE = 1.88 × 10^–2^). Nonetheless, high estimates for p indicate that the data set provides a representative picture of the aggregation within SAMPA.

**Figure 6:**
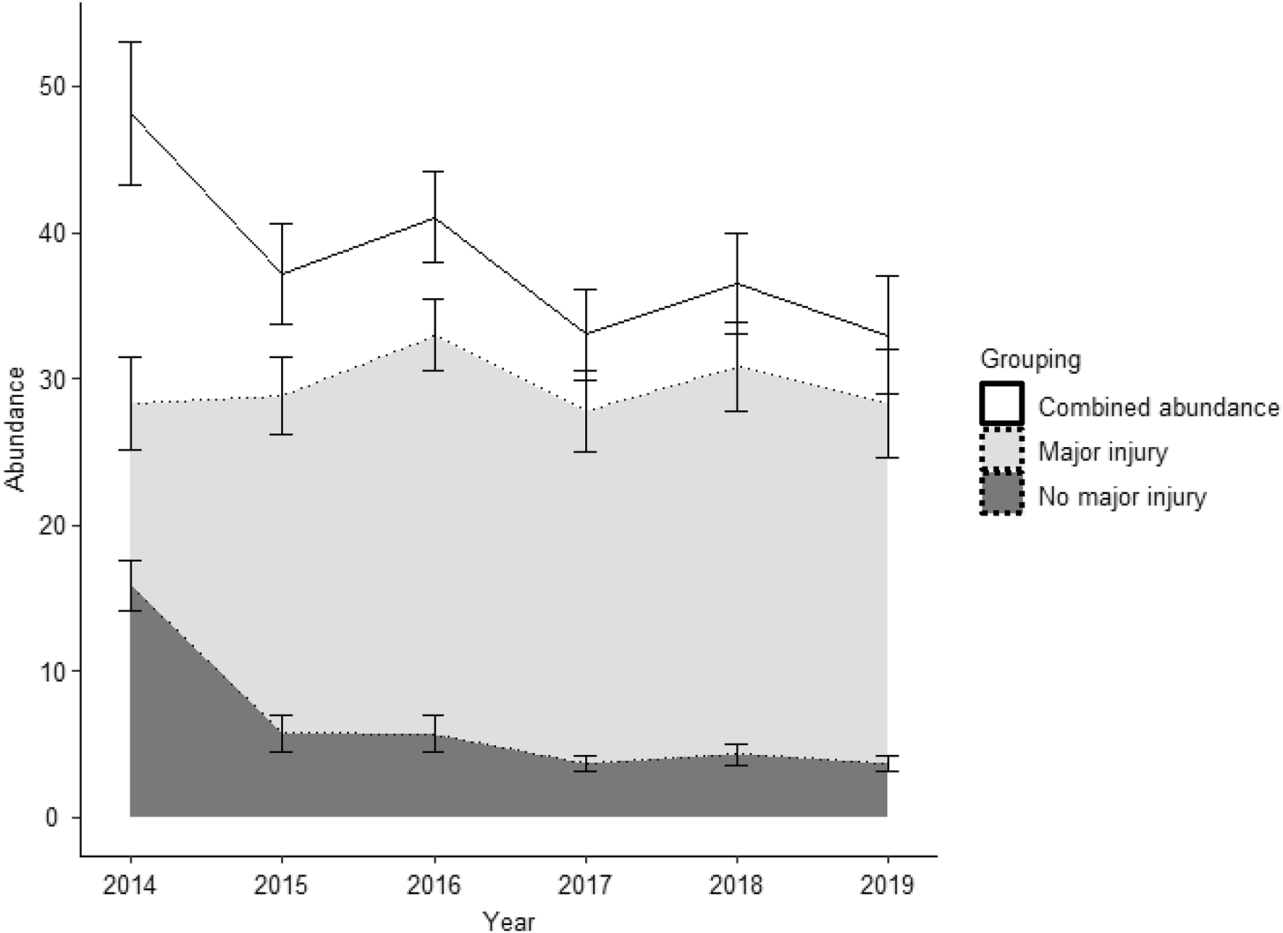
Area chart showing abundance of sharks within the study area (with SE) calculated as a derived parameter from POPAN modelling across the years 2014–2019. Total combined abundance of all sharks (white) declines across the study period. Abundance for male sharks with no major injuries (black) appears to drop, whilst abundance for male sharks with major injuries (grey) appears relatively stable. Note that annual abundance is derived from the estimated parameters N, Phi, p and pent and differences in annual abundance among groups may not match differences in total abundance, N. Female sharks within injury groupings are not included due to an average abundance of 0.

Abundance across years (2014–2019) was modelled as a derived result from the POPAN model (Fig. 6). Additionally gross N* population was modelled as a derived POPAN result. The gross population was estimated as 60.25 (SE = 0.74) males with major injuries and 50.19 (SE = 4.99) males with no major injuries. 3 (SE = 0) females with major injuries were estimated and 1 (SE = 0) female with no major injuries, totalling 114 individuals.

**Figure 7:**
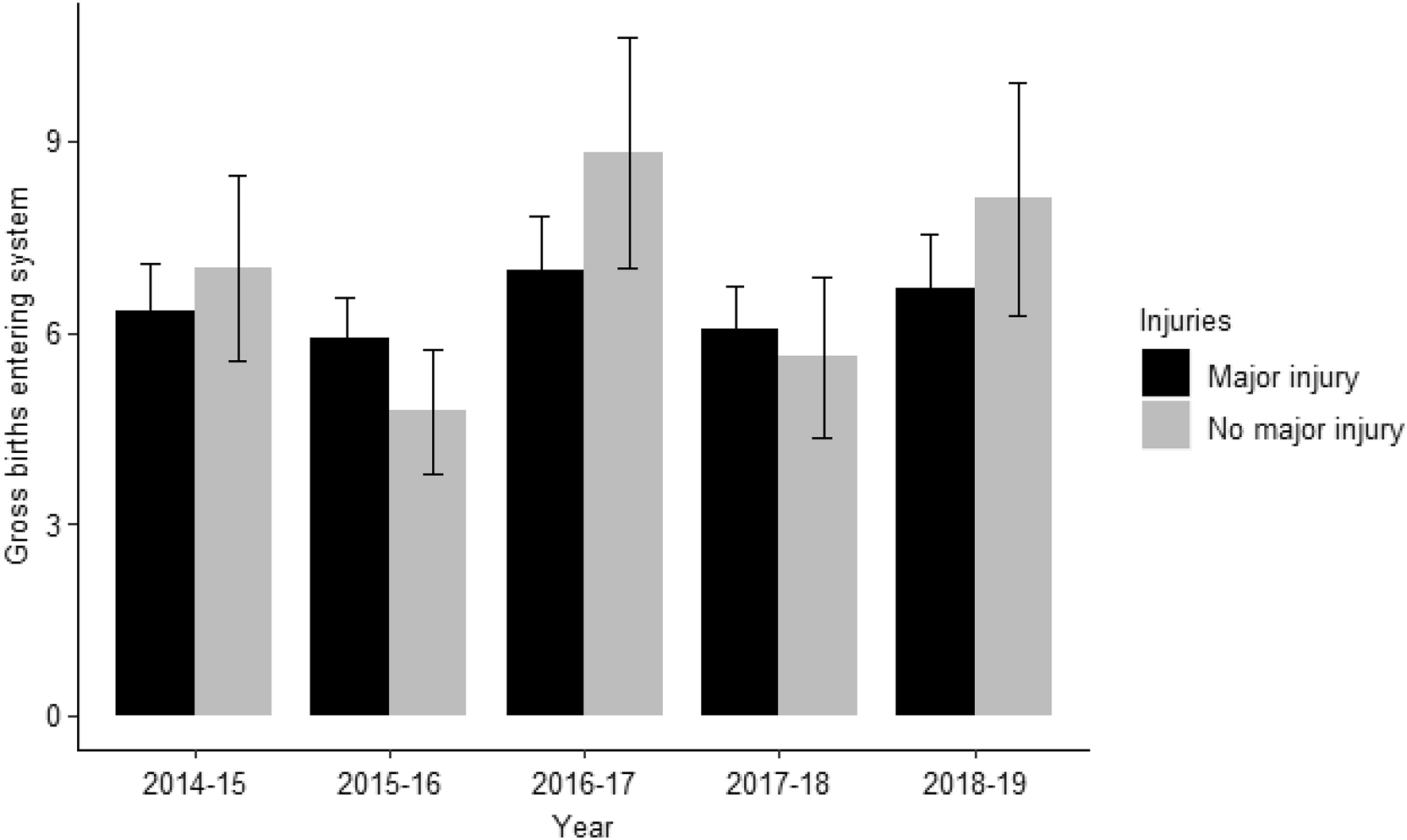
Gross amount of individuals (with SE) with a major injury (black), and no major injury (grey) entering SAMPA between years i and i + 1 with standard error. Gross ‘births’ are calculated as a derived POPAN parameter.

Both Net and Gross births (Fig. 7) into SAMPA were calculated as derived POPAN results. Net births into the population (between times i and i + 1) was calculated to be 2.86 × 10^–11^ (SE = 3.37 × 10^–9^) for females with major injuries and 5.75 (SE = 6.32 × 10–1) for males with major injuries. For individual with no major injuries, Net births was calculated to be 9.53 × 10^–12^ (SE = 6.31 × 10^–1^) for females and 3.23 (SE = 3.55 × 10^–1^) for males. Models predicting probability of entry (PENT) based on sex were found to be the best fit to sightings data. Male sharks had a considerably higher PENT value (0.10, SE = 0.01) than females (3.54 × 10^–17^, SE = 2.82 × 10^–15^).

### Lagged identification rate

Model H (Supplementary Table 2) was the best-fit model for whale sharks with and without major injuries, which included parameters for emigration, reimmigration and mortality or permanent emigration. For whale sharks with major injuries, the LIR dropped steadily as the time lag increased (Fig. 5b), never quite reaching zero, suggesting some individuals may stay within the site over long periods of time. Contrastingly, the LIR for whale sharks without major injuries was very high (0.125) after 1 day, then dropped off quickly as the time lag increased, reaching zero after ~ 4400 days or ~ 12 years (Fig. 5b). Parameter estimates showed that sharks with major injuries had a mean initial residency of 46.5 (SE = 10.8) while non-injured sharks had a mean initial residency of 69.9 (SE = 29.8) days. Sharks with major injuries spent a mean of 28.5 (SE = 7.7) days outside the study area, whilst non-injured sharks spent 18.1 (SE = 14.8) days outside the study area before re-entering the study site. Mean mortality was estimated at 0.0004 (SE = 0.0002) for sharks with major injuries and 0.001 (SE = 0.001) for non-injured sharks, indicating an apparent survival of 0.85 and 0.49 year^−1^ respectively. The model also estimated daily abundance at the site, with 13.7 (SE = 1.7) sharks with major injuries and 9.0 (SE = 1.9) non-injured sharks present.

### Association between residency and major injury on arrival

**Figure 8:**
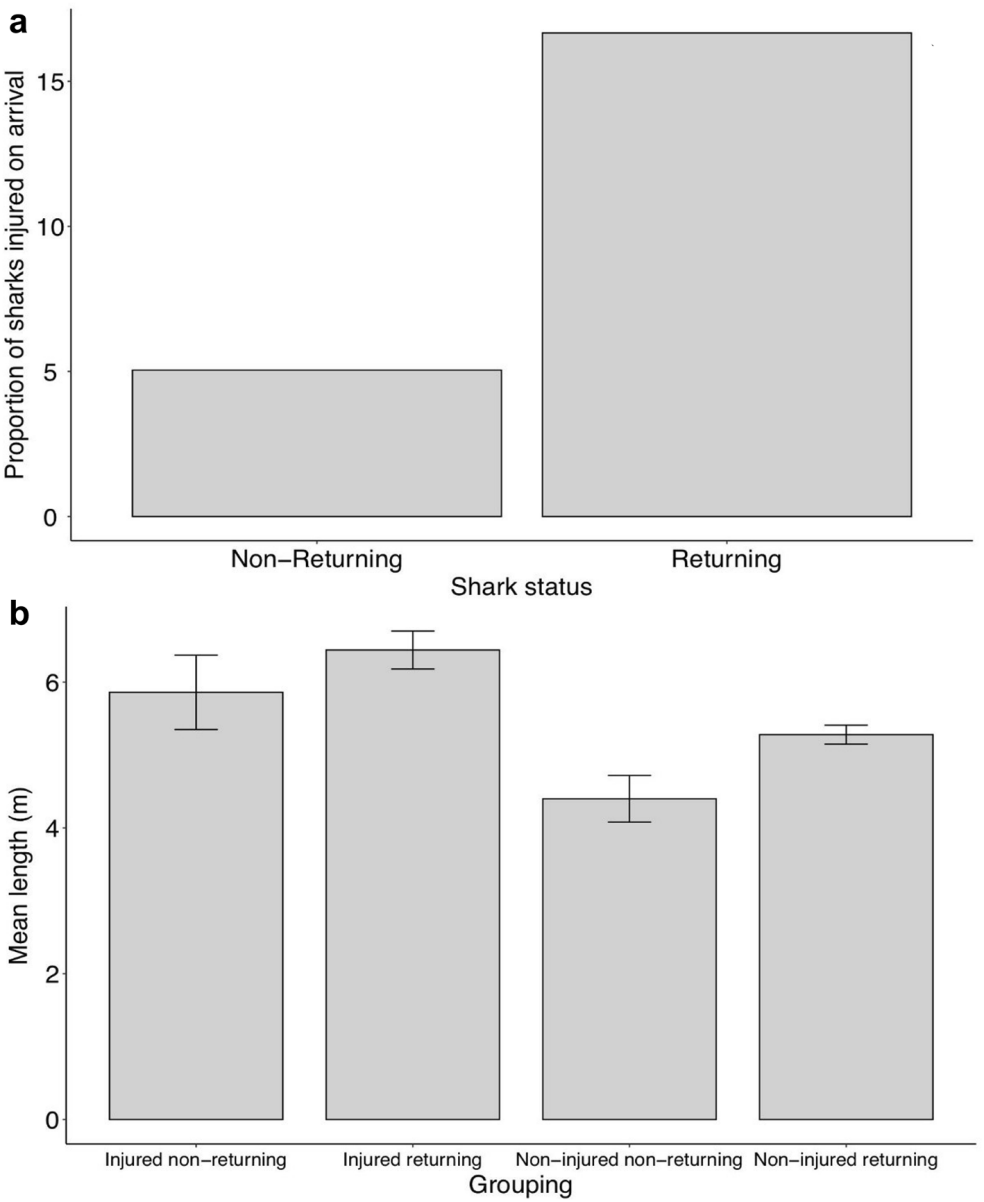
(**a**) Proportion of individual sharks with major injuries on arrival that return the following year vs. non-returning individuals between the years of 2006–2019. (**b**) Mean length (with SE) of resident and transient whale sharks with and without major injuries for the years 2006–2019.

We found a significant association between major injury on entry (first time the individual is recorded in SAMPA) and shark residency status (p < 0.001, DF = 1, χ^2^ = 13.83) (Fig. 8a). A higher proportion of returning sharks were injured upon entry (17%) compared to transient sharks (5%) (Fig. 8a).

Shark length on entry was found to be significantly related to both returning (F = 6.75, DF = 1, p = 0.01) and initial major injury status (F = 18.63, DF = 1, p < 0.001) (Fig. 8b). Post-hoc testing indicated that mean length for returning injured (6.44 m, SD = 1.05) and transient injured groups (6.65 m, SD = 0.21) were significantly higher than returning non-injured (5.27 m, SD = 1.4) and transient, non-injured (4.4 m, SD = 1.2) sharks.

## Discussion

We modelled the population dynamics of a unique aggregation of juvenile whale sharks within the South Ari Atoll Marine Protected Area (SAMPA). Our analyses showed that the presence of major injuries was associated with a higher survival probability (represented by lower emigration) and more time spent in SAMPA. Our results suggest a decline in abundance since 2014 for the SAMPA aggregation—potentially due to avoidance of the area or decline in meta-population. We suggest conservation measures targeted at vessel traffic to reduce the negative impact of tourism on this charismatic species. This will also protect the sustainable income jobs that local whale shark aggregations create in the Maldives.

Whale sharks are a highly mobile species known to form seasonal aggregations throughout their range. Year-round residency is uncommon, with a few examples such as Mafia Island, Tanzania^58^, Ningaloo^59^ and provisioned whale sharks in Oslob (Philippines^60^). Our findings support the hypothesis that the SAMPA aggregation contains both returning and non-returning individuals^34^. Individual sharks are sighted throughout the year, suggesting they remain in the general area. This is supported by our modified maximum likelihood approach, which predicted both injured and non-injured sharks spending longer within SAMPA than outside it—as has been reported for Mafia Island^61^ and Oslob^62^. The relatively small size of individuals, ranging from 2.5 to 8.0 m, supports the current view of SAMPA as a developmental habitat for juvenile whale sharks. Photo Identification has confirmed that whale sharks move between Maldivian Atolls throughout the year. The fact that individual sharks return to SAMPA highlights the importance of the area for this Endangered Species with high levels of exploitation in the Indo-Pacific^63^.

We calculated a general decline in abundance of Whale Sharks since 2014 (Fig. 6). Our annual abundance estimates can be interpreted as the total number of whale sharks present in SAMPA that could be sighted at one time. This does not include previously identified individuals that are not present. Therefore, the declining abundance trend we observe may represent a true reduction in shark numbers, or the influence of external factors linked to migration and regional, inter-atoll distribution. In either case, a decline in whale sharks accessing SAMPA each year appears to be present.

As a developmental habitat, the main source of new individuals in SAMPA aggregation is likely immigration. Probability of entry into the aggregation was estimated at different rates based on sex (Males 0.10, SE = 0.01 and Females 3.54 × 10^–17^, SE = 2.82 × 10^–15^). The reason for this sexual segregation is unclear. However, most whale shark aggregations globally are dominated by juvenile males^35,64,65^.

Estimated LIR indicated that there are *ca*. 14 whale sharks with major injuries and *ca*. 9 without injuries on any given day within SAMPA. These estimates are comparable to other coastal aggregations assessed using the same methods. Examples include Saudi Arabia’s Red Sea (~ 21 individuals)^66^, Southern Leyte in the Philippines (~ 16 individuals)^67^, and Oslob, Philippines (~ 19 individuals)^62^. It is considerably lower though than other aggregations like Qatar (124^68^), Donsol, Philippines (50^69^), Honda Bay, Philippines (41^25^), or Mozambique (51^61^).

Previous studies by Speed et al.^27^ and Lester et al.^28^ have suggested that the presence of major injuries has no significant impact on apparent survival of the seasonal Ningaloo aggregations of whale sharks. Lester et al.^28^ found an apparent survival of 0.88 in sharks with major injuries compared to 0.82 for non-injured sharks at the seasonal Ningaloo Reef aggregation. Our model selection approach suggests that there is a significant difference between survival of injured and non-injured sharks. We report a substantially lower apparent survival (likely representing higher levels of emigration) of non-injured sharks in the SAMPA aggregation compared to those with major injuries (Fig. 5a) (mean value across years for injured individuals = 0.80, compared to mean values for non-injured sharks = 0.20). This is unlikely to be confounded by sighting probability, as we predicted a high probability of sighting both injured and non-injured sharks.

Complementing the POPAN results, LIR modelling predicted whale sharks with major injuries to have a high probability of returning over time with seasonal peaks, and a low mortality/permanent emigration (~ 0.15 year^−1^). This suggests individuals with major injuries might reside within SAMPA, or in close proximity of the site, for long periods of time. Contrastingly, the LIR for non-injured individuals drops sharply over time, eventually reaching zero. Permanent emigration or mortality for non-injured sharks was high (0.52), suggesting these animals are likely more transient to SAMPA on average than injured individuals. This higher rate of emigration or mortality predicted by LIR is consistent with the POPAN results. High estimated residency times (69.9 days) within SAMPA suggests that some non-injured sharks initially remain in the aggregation for a considerable time. However, they eventually leave the site at higher rates than injured sharks, as indicated by the higher mortality and permanent emigration rate. This can be explained by the heterogeneity in the residency patterns of non-injured whale sharks at SAMPA. Some individuals are only seen once (~ 50%) and some individuals are resighted over many years. The impact of this heterogeneity on LIR modelling has been noted in other taxa (e.g. marine turtles^70^). Modelling of days spent outside of the study site is difficult given the lack of absence data and should therefore be cautiously interpreted.

There is no definitive method to determine if injuries are acquired within, or outside, SAMPA using the current data. Whale sharks are known to swim large distances in 1 day (26 km day^−1^^22^) with average speeds ranging from 1.17 to 3.19 km h^−1^^71^. SAMPA is a small area spanning 42 km^2^ (with a 1 km width), so it is likely that whale sharks leave SAMPA and return after a period of time. Major injuries identified in this dataset are likely the result of collisions with small to medium sized vessels (i.e. gulf crafts and speedboats) which are present within, and outside, of SAMPA. We calculated the average time from last sighting of an individual to the first report of a new injury to be 128 days. Given the LIR calculated residency rates of 46.5 and 69.9 days for sharks with major and no major injuries, respectively, this indicates some injuries may be acquired outside of SAMPA, likely from faster moving inter-atoll transport.

We propose that sharks are acquiring sublethal injuries, resulting in higher apparent survival rates due to reduced emigration. Lower apparent survival in non-injured individuals could indicate permanent emigration from the aggregation. Similar interpretations of apparent survival have been drawn in studies of cetaceans^72^. Sharks arriving in the area with major injuries spend longer in SAMPA than those without. This may be due to a suitable recovery environment in the developmental habitat^73^, such as warmer waters, few natural predators, and an abundance of food^31,74,75^. Supporting this, we found a significant association between the presence of a major injury on first recorded entry of sharks into SAMPA and returning status (Fig. 8a). These injuries most likely occur outside of SAMPA. This implies that sharks are more likely to return or remain in SAMPA if they have an initial major injury when first arriving. This supports the idea that sharks may stay in SAMPA due to an ideal recovery environment.

Whale sharks likely reside in SAMPA as a developmental habitat. An alternative explanation may be that returning sharks have increased probability of acquiring injuries. In their life history, their period of residency in SAMPA may be a time when individuals are intensely subjected to tourist encounters and vessel traffic. This may lead to an accumulation of injuries in returning sharks.

Work by Quiros, Haskell, and Araujo et al.^76–78^ has indicated that previously injured sharks are less likely to display avoidance behaviour, with longer encounter times. We found that larger, more mature individuals are more likely to have a major injury on entry into the area than smaller, younger animals (Fig. 8b). Larger individuals have likely been injured elsewhere before entering SAMPA, and return more than uninjured sharks (Fig. 8b). Our results may indicate that differences in life history, or behavioural ecology, can result in varying levels of injury accumulations, as found in the Southern Leyte, Philippines aggregation^78^. Such differences are also known within other species of sharks, such as lemon sharks (*Negaprion brevirostris)*^79^. Additionally, LIR analysis (Fig. 5b) found non-injured sharks are more often resighted after shorter intervals, whilst injured sharks are more likely to be resighted after longer intervals. This indicates that injured sharks likely have higher rates of residency and site fidelity.

Providing a conclusive assessment of the SAMPA whale shark aggregation, and where they may be acquiring injuries, would require an in-depth study of individual movement and its overlap with boat traffic and other potential sources of anthropogenic injuries. This could be achieved using acoustic^58^, satellite^25^, or multiaxial^80^ telemetry to study movement of individual sharks. Such technology is a powerful tool for understanding how best to protect shark populations, however there are significant barriers including cost and negative public opinion surrounding tagged megafauna welfare.

Injuries require significant resources to heal^81–83^. Whale sharks have a tightly constrained energy budget due to their behavioural ecology and physiology^84^. The high demand of energy towards healing of major injuries during a critical growth phase (such as the juvenile SAMPA aggregation) may cause disruption to resource allocation for other metabolic processes, in turn reducing fitness^82,85–87^. If large energy demands from injuries are combined with a chronically stressed population it is likely to have detrimental effects on fitness^81,85,88,89^.

SAMPA whale sharks receive a considerable amount of tourist attention^40^. Voluntary whale shark tourist encounter guidelines, published by the Maldivian government, prohibit touching, obstructing, and crowding of animals^90^. However, this code of conduct is known to be breeched frequently^91,92^. This may lead to high levels of chronic physiological stress^93–96^, and increased energy expenditure^97^. When guidelines are broken and whale sharks are approached closely, or touched by snorkelers, extreme behavioural changes are frequently demonstrated^77,98,99^. This may be indicative of an acute stress response, associated with large energetic demands^93^. Frequent high energy behaviours are thought to interrupt normal behaviours such as thermoregulation and foraging and may have a detrimental impact on growth, reproduction, immune function, and survival^93,100,101^. Furthermore whale sharks may actively avoid tourist interactions, resulting in exhaustive exercise, which is known to exacerbate the detrimental effects of injuries^77,99,102^.

We found that whale sharks with major injuries may reside for longer in SAMPA, potentially resulting in a cumulative effect of these stressors. The combination of physiological stress from injuries and behavioural stress resulting from tourist interactions are likely to have dire consequences on the declining Maldivian shark population. Improving whale shark protection policies within SAMPA will reduce major injuries, and promote welfare and fitness of this juvenile aggregation. Given the year-round occurrence of whale sharks in SAMPA, it is imperative that management strategies are devised to minimise the impact of human activities at the site. Injuries from vessels can be managed for in areas of high occurrence. Interventions have been employed across global whale shark aggregation sites to minimize the chance of collision with sharks. Examples include speed limits for vessels and separation distances between vessels and sharks (see Lester et al.^28^).

SAMPA is in a unique position due to the high level of local knowledge about the preferred locations and distribution of sharks around the MPA. This allows for targeted management efforts. Maintaining the health of the SAMPA aggregation will require further measures to prevent injuries from boat strikes. We suggest the most effective first step would be to transition current voluntary encounter guidelines into enforceable regulations. A draft management plan for SAMPA is being developed in consultation with the tourism industry and local communities. Proposed regulations, if enforced, could greatly reduce injuries by simple measures, such as reducing vessel speed when traveling within the MPA. A transition to enforceable regulations is likely to be supported by all stakeholders. Given the importance of whale sharks to the local economy, there is a high degree of investment in shark health and conservation.

Limiting numbers of vessels within the MPA by the use of a zoning system, and a scheduling rota of visitation based on vessel type, would also prove valuable. Use of an electronic monitoring system, as proposed by Speed et al.^103^ may aid in monitoring vessel compliance. Furthermore, accredited training for guides and vessel crews as a requirement for licence to enter the MPA would raise the level of awareness among operators. A standard industry-wide briefing for tourists delivered before the excursion would also relieve diver pressure on the guides and tour operators. Based on current MPA rules, and dive sites in the area, multi-stakeholder consultation would be essential in order to develop an effective reduction strategy for vessel and shark collisions.

## Supplementary Information

Supplementary Information.
