# Supplementary material for: The impact of injury on apparent survival of whale sharks (Rhincodon typus) in South Ari Atoll Marine Protected Area, Maldives
Source: Sci Rep. 2021 Jan 13;11:937. doi: 10.1038/s41598-020-79101-8 (PMC7806644; doi:10.1038/s41598-020-79101-8)
Supplement: Supplementary file 1 — Supplementary Information. [file 41598_2020_79101_MOESM1_ESM.pdf]

The impact of injury on apparent survival of whale sharks (*Rhincodon typus*) in the South Ari Atoll Marine Protected Area, Maldives

Jessica Harvey-Carroll*[1,2], Joshua D. Stewart[3], Daire P Carroll [1,5,6], Basith Mohamed[1], Ibrahim Shameel[1], Irthisham H. Zareer[1] , Gonzalo Araujo[7], Richard Rees[1]

[1] Maldives Whale Shark Research Programme (MWSRP), South Ari Atoll, Maldives

[2] School of Psychology and Neuroscience, University of St Andrews

[3]The Manta Trust, Dorchester, UK

[5]The James Hutton Institute

[6]The University of Warwick, School of Life Science

[7]Large Marine Vertebrates Research Institute Philippines, Cagulada Compound, Jagna, 6308, Bohol, Philippines.

Corresponding author: Jessica Harvey-Carroll,

jessica-lily@maldiveswhalesharkresearch.org

Supplementary

Table 1. Model selection weighting using the AICc of the top 20 most parsimonious POPAN models.

No.	Model	No. of parameters	AICc	Delta AICc	Weight	Deviance
51	Φ(~time + injury)p(~injury + sex)PENT(~sex)N(~1)	12	342.5738	0	2.82E-01	-149.393
49	Φ(~time + injury)p(~injury + sex)PENT(~1)N(~1)	11	343.9888	1.415028	1.39E-01	-145.741
41	Φ(~time + injury)p(~injury)PENT(~1)N(~1)	10	344.4882	1.914379	1.08E-01	-143.026
83	Φ(~time + injury + sex)p(~injury + sex)PENT(~sex)N(~1)	13	344.7607	2.186944	9.46E-02	-149.465
43	Φ(~time + injury)p(~injury)PENT(~sex)N(~1)	11	345.8991	3.325298	5.35E-02	-143.831
73	Φ(~time + injury + sex)p(~injury)PENT(~1)N(~1)	11	345.9301	3.356318	5.27E-02	-143.8
81	Φ(~time + injury + sex)p(~injury + sex)PENT(~1)N(~1)	12	345.9784	3.40464	5.14E-02	-145.988
33	Φ(~time + injury)p(~1)PENT(~1)N(~1)	9	347.3312	4.757371	2.62E-02	-137.989
75	Φ(~time + injury + sex)p(~injury)PENT(~sex)N(~1)	12	347.6429	5.06908	2.24E-02	-144.324
55	Φ(~time + injury)p(~injury + sex)PENT(~time + sex)N(~1)	16	347.8031	5.229277	2.07E-02	-153.33
52	Φ(~time + injury)p(~injury + sex)PENT(~sex)N(~group)	15	348.4141	5.840283	1.52E-02	-150.394
65	Φ(~time + injury + sex)p(~1)PENT(~1)N(~1)	10	348.7055	6.131669	1.32E-02	-138.809
53	Φ(~time + injury)p(~injury + sex)PENT(~time)N(~1)	15	348.987	6.413193	1.14E-02	-149.821
35	Φ(~time + injury)p(~1)PENT(~sex)N(~1)	10	349.0257	6.451949	1.12E-02	-138.489
45	Φ(~time + injury)p(~injury)PENT(~time)N(~1)	14	349.2015	6.627705	1.03E-02	-147.304
50	Φ(~time + injury)p(~injury + sex)PENT(~1)N(~group)	14	349.7488	7.174955	7.81E-03	-146.757
42	Φ(~time + injury)p(~injury)PENT(~1)N(~group)	13	349.8973	7.323464	7.25E-03	-144.328
87	Φ(~time + injury + sex)p(~injury + sex)PENT(~time + sex)N(~1)	17	350.0802	7.506388	6.62E-03	-153.401
34	Φ(~time + injury)p(~1)PENT(~1)N(~group)	12	350.4857	7.91185	5.40E-03	-141.481

Table 2. Model results for modified maximum likelihood methods using parameters to test for population closure, mortality and permanent emigration, re-immigration and residency as preset in program SOCPROG 2.9 (Whitehead, 2009). N = population size; QAIC: quasi-Akaike information criterion.

Model Name	Parameters	ΔQAIC Major Injuries	ΔQAIC No Major Injuries
A	Closed (1/a1=N)	12087.4256	6204.9357
B	Closed (a1=N)	12087.4256	6204.9357
C	Emigration/mortality (a1=emigration rate; 1/a2=N)	281.0753	3.3815
D	Closed: Emigration + reimmigration (a1=emigration rate; a2/(a2+a3)=proportion of population in study area at any time)	45.8927	6038.6056
E	Emigration/mortality (a1=N; a2=Mean residence time)	281.0753	3.3814
F	Emigration + reimmigration + mortality	227.6205	2.5629
G	Emigration + reimmigration (a1=N; a2=Mean time in study area; a3=Mean time out of study area)	45.8926	5.3814
H	Emigration + reimmigration + mortality (a1 = N; a2 = mean time in	**0**	**0**

	study area; a3 = mean time out of study area; a4 = mortality rate)		
